# Who killed my dog? Use of forensic genetics to investigate an enigmatic case

**DOI:** 10.1007/s00414-020-02388-9

**Published:** 2020-08-11

**Authors:** Mariana Roccaro, Carla Bini, Paolo Fais, Giuseppe Merialdi, Susi Pelotti, Angelo Peli

**Affiliations:** 1 Department of Veterinary Medical Sciences, University of Bologna, Ozzano dell’Emilia, Italy; 2 Department of Medical and Surgical Sciences, Unit of Legal Medicine, University of Bologna, Bologna, Italy; 3 Istituto Zooprofilattico Sperimentale della Lombardia e dell’Emilia–Romagna “Bruno Ubertini”, Bologna Unit, Bologna, Italy

**Keywords:** Forensic genetics, mtDNA, Cytochrome b, Hair, Dog, Animal attacks

## Abstract

Genetic testing of animal biological material has become a valuable tool in forensic investigations, and it is successfully used to identify unknown crime perpetrators, to unmask food frauds, or to clarify cases of animal attacks on humans or other animals. When DNA profiling is not possible due to inadequate amounts of nuclear DNA, mitochondrial DNA (mtDNA) testing is the only viable alternative, as in the case of shed hair samples. In this case, a dog was allegedly killed by wild animals while being hosted in a boarding house. Extraneous hair fragments recovered from the dog’s mouth and paws were subjected to genetic analysis: the cytochrome *b* gene located on mtDNA was amplified and sequenced in order to determine the species responsible for the killing. The mtDNA analysis provided evidence that the dog was killed by other dogs, thus unmasking a false wild animal attack and putting the case in an entirely different perspective.

**Electronic supplementary material:**

The online version of this article (10.1007/s00414-020-02388-9) contains supplementary material, which is available to authorized users.

## Introduction

Genetic testing of animal biological material has become a valuable tool in civil and criminal forensic investigations [1–3]. Animals can help linking victims and/or suspects to a crime scene [4–7], but they can also be directly involved in an investigation either as victims or offenders, as in cases of property damage or attacks on humans or other animals [8–13].

Genetic testing can be performed on a wide variety of biological materials, such as blood, faeces, urine, semen, bone, skin, hair, fur and other tissues.

Nuclear DNA testing allows individual identification through STR (short tandem repeat) or SNP (single nucleotide polymorphism) profiling [14–16]. However, if the amount and/or quality of nuclear DNA in the sample is inadequate, the more abundant and resistant mitochondrial DNA may be suitable for genetic analysis. This is the case with shed hair samples, which often do not contain follicular material [17–20].

MtDNA is primarily used for species identification by the analysis of the cytochrome *b* gene (*CytB*), the most common locus on mtDNA used for species determination [21–25]. Other useful regions are the cytochrome oxidase subunit I gene (COI) and the 16S ribosomal RNA gene [26–28].

MtDNA sequencing can also target the two hypervariable regions (HV1 and HV2) within the D-loop and several studies have been published on the canine mtDNA D-loop, although an exhaustive canine mtDNA population database is still unavailable [29–34]. However, the potential value of this method in forensic analysis can be considerable for the exclusion of an individual dog as a source of evidence [19, 30].

In this case, we applied genetic analysis of *CytB* on animal hairs in order to determine the species of the animal(s) responsible for killing a dog.

## Case history

An 8-year-old female Jack Russel Terrier hosted in a dog boarding house was found dead in the courtyard. The net of the kennel where the dog was held was broken, as if it had been pulled from the inside. The veterinarian who was called to the scene hypothesised that the cause of death was attributable to traumatic injuries inflicted by wild animals, such as foxes or nutrias. The tenant of the dog boarding house owned three Hovawart dogs.

The dog’s carcass was left at ambient temperature (average daily temperature 7.0 °C) for 18–20 h and then stored at − 18 °C pending post mortem examination, which was performed 1 month later at the diagnostic section of the Istituto Zooprofilattico Sperimentale of Lombardia and Emilia-Romagna located in Bologna.

Our team was contacted by the dog’s owner in order to assist with the necropsy and to possibly identify the animal(s) responsible for the killing.

At the external inspection, the dog appeared in good nutritional condition.

**Fig. 1:**
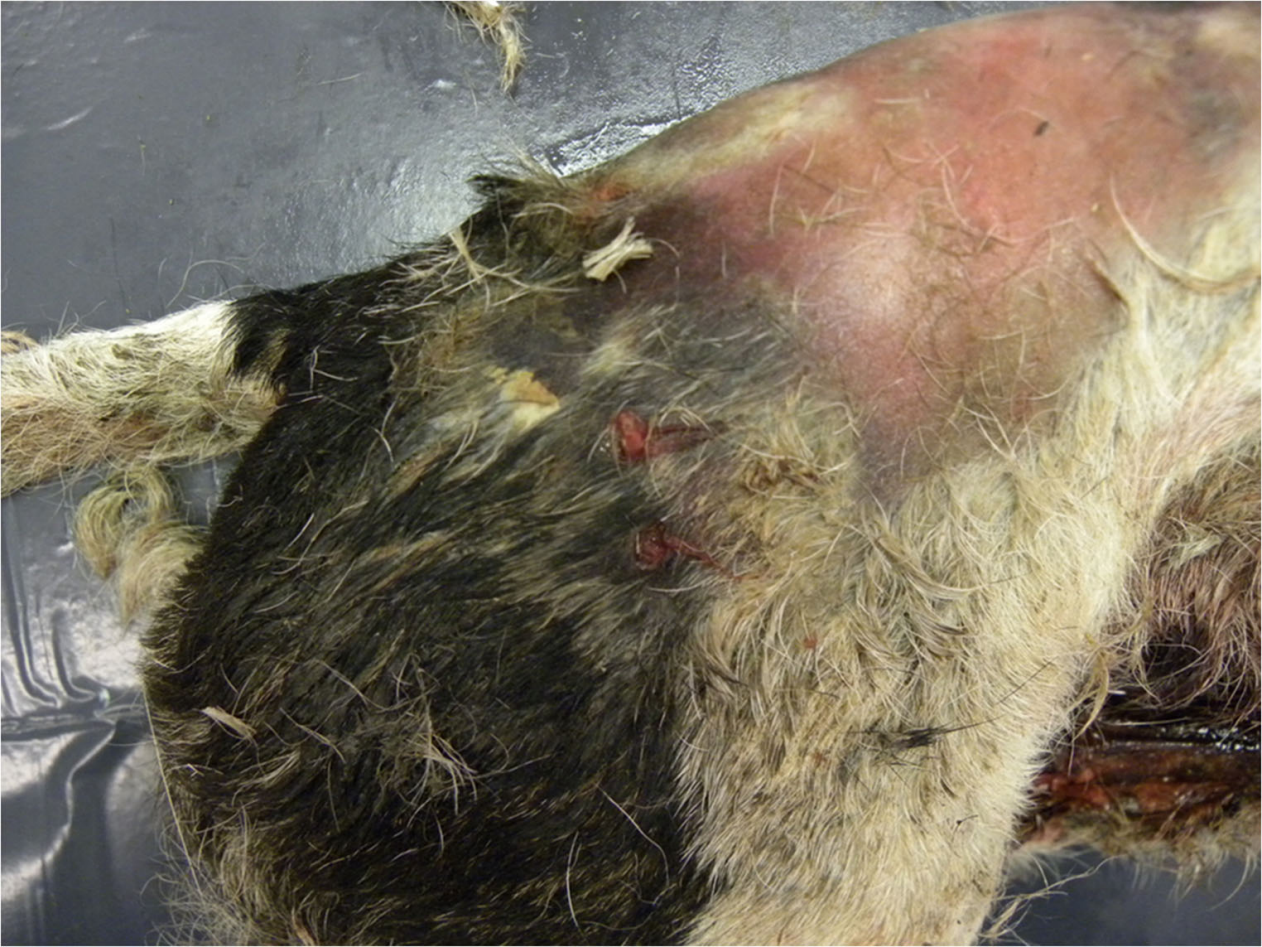
Bite marks on the victim’s right thigh

The haircoat was blood-stained and 14 wounds, 7–10 mm in size, were observed in various areas of the body: one on the right side of the nape, one on the dorsal side of the neck and three more on the ventral side, one on the left shoulder, one on the left thorax and two more behind the costal arch, two on the right thigh and three in the inguinal region. These wounds were oval with sharp margins and were therefore attributed to bite marks (Fig. 1).

An additional and larger wound, 100 mm long and 25 mm wide, was observed in the lumbar region.

**Fig. 2:**
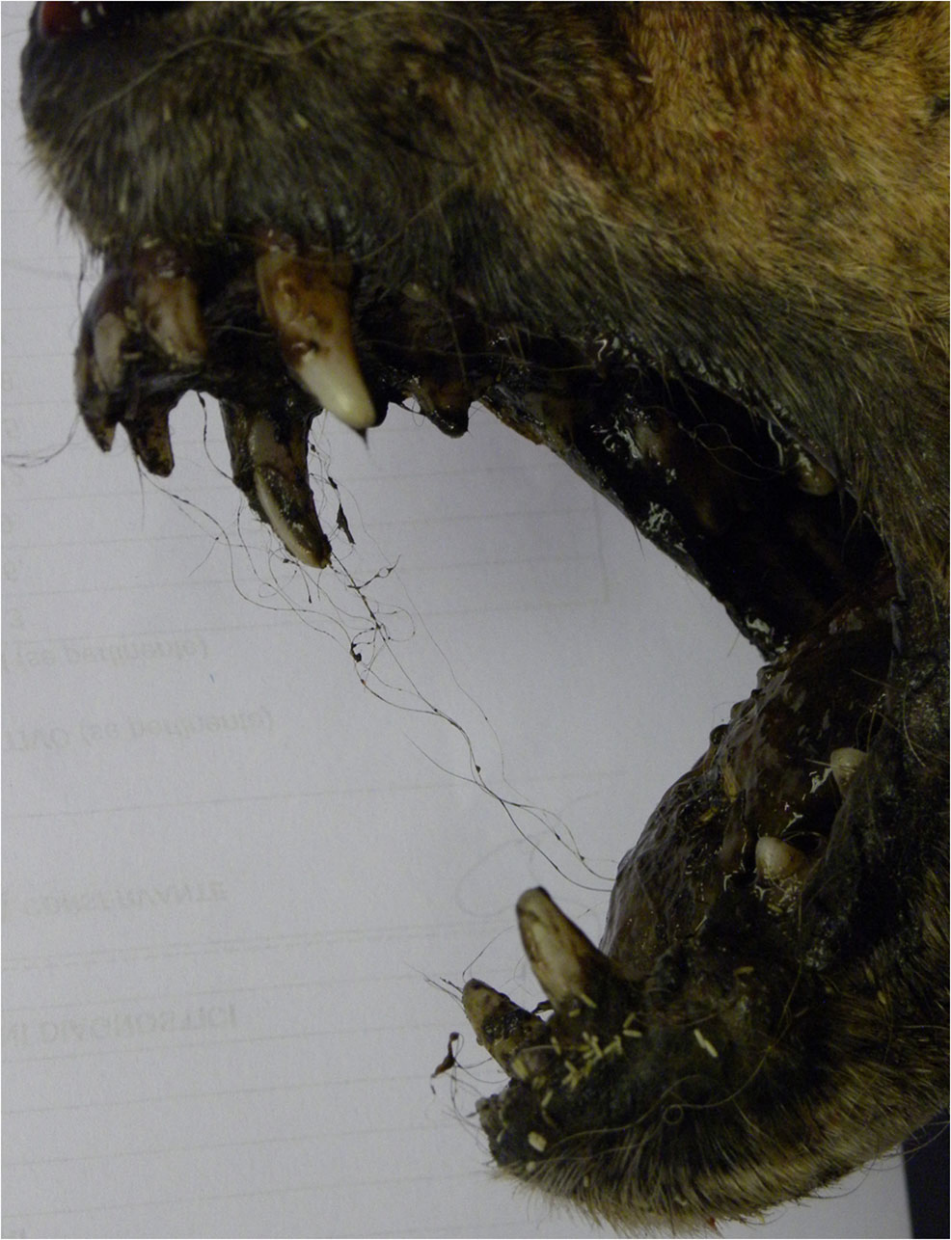
Hairs caught between the victim’s teeth

**Fig. 3:**
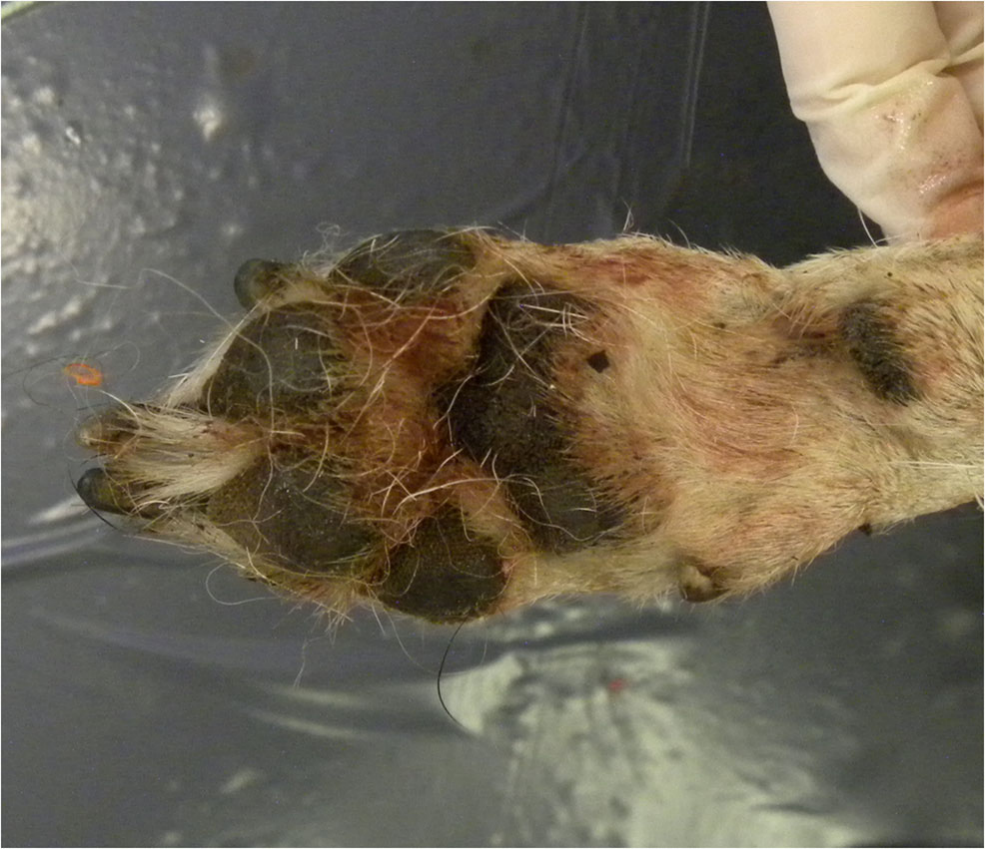
Hairs entangled in the victim’s right forepaw

The inspection of the oral cavity revealed the presence of numerous hairs, caught between the teeth (Fig. 2); other hairs were entangled in the paws (Fig. 3). The length (50–100 mm) and colour (some light/fair, others dark) of the hairs, in addition to the unnatural location where they were found, were incompatible with those of the dog examined. These hairs were therefore sampled for genetic analysis.

When skinned, sub-dermal haemorrhages were observed behind the wounds, which extended to the muscles on the left side of the neck and the thorax, where the haemorrhagic infiltration was massive. Upon opening the abdomen and the thoracic cavity, a small amount of free blood was observed. Despite the poor state of preservation, all organs in the abdominal and thoracic cavity were found to be normal on gross observation. The abdominal wall was perforated at the wounds detected behind the costal arch. Similarly, the intercostal muscles and the pleura were perforated between the eight and the ninth rib. Moreover, the fifth rib was fractured. The signs of vital reactions in the affected tissues allowed to determine that the lesions observed where inflicted while the victim was still alive.

Cause of death was ascribed to a fatal pneumothorax resulting from multiple penetrating bite wounds to the thorax. The post mortem examination thus provided conclusive evidence of a fatal attack, perpetrated by one or more animals. The presence of numerous hairs, caught between the teeth and the paws of the victim, was indicative of a fight.

## Materials and methods

The DNA analysis was performed in an ISO 9001:2015 certified laboratory. The laboratory does not routinely conduct non-human DNA testing; for this casework, all the analyses were performed following the recommendations set out by the International Society for Forensic Genetics (ISFG) regarding the use of non-human DNA in forensic genetic investigations [35].

Hairs showing different characteristics were selected for the analysis; the longest and the most intact ones were preferred. Several fragments of four hair samples (three collected from the dog’s mouth and one from the paw) were firstly decontaminated in order to eliminate any exogenous DNA by following the protocol of Jehaes et al. [36].

DNA was extracted using the QIAamp DNA Investigator kit (Qiagen) following the protocol of the manufacturer for the “Isolation of Total DNA from Nail Clippings and Hair” with an overnight incubation at 56 °C and a final DNA elution in 20 μl of buffer ATE.

The isolated DNA was analysed following the protocol of Hsieh et al. [22] to amplify the *CytB* gene region using the primers L14724 (5′-CGAAGCTTGATATGAAAAACCATCGTTG-3′) and H15149 (5′-AAACTGCAGCCCCTCAGA ATGATATTTGTCCTCA-3′).

PCR amplification was performed in 25 μl of reaction volume containing 2.5 μl of 10X PCR of Buffer II, 1.5 mM of MgCl2, 0.2 mM of each dNTP, 0.25 μM of each primer, 1.25 U of AmpliTaq Gold DNA Polymerase (Thermofisher Scientific) and 10 μl of template DNA. Amplification was conducted on a 9700 Thermal Cycler (Applied Biosystems) at the following conditions: 95 °C for 11 min, 35 cycles of 94 °C for 30 s, 50 °C for 45 s and 72 °C for 45 s and a final extension of 72 °C for 7 min. PCR products were purified using the ExoSAP-IT PCR clean-up protocol (USB Corporation).

Cycle sequencing was performed using the Big Dye Terminator Cycle Sequencing Kit v.1.1 (Applied Biosystems) on a 9700 Thermal Cycler (Applied Biosystems) at the following conditions: 96 °C for 1 min, 25 cycles of 96 °C for 45 s, 50 °C for 20 s and 72 °C for 4 min. Forward and reverse sequences were obtained using both primers L14724 or H15149. The cycle sequencing reaction contained 3 μl of purified PCR product, 2 μl BDT v1.1 Ready Reaction Mix, 2 μl BDT v1.1 5X Sequencing Buffer, 0.5 μM sequencing primers and water grade to reach a total reaction volume of 20 μl.

PCR sequencing products were separated through CE using a POP4 polymer on ABI310 Genetic Analyzer (Applied Biosystems) and analysed by the Sequencing Analysis Software v 5.2 (Applied Biosystems). Negative controls were included in each extraction and PCR run to monitor for contamination, as well as a human DNA sample as PCR positive control.

The sequences were aligned and compared with the species-specific *CytB* sequences available on GenBank® by using the BLAST (Basic Local Alignment Search Tool).

## Results and conclusion

Single source mtDNA sequences were obtained from all four hair samples and data from negative and positive controls excluded exogenous DNA contamination.

The electropherograms of a forward and reverse reference sequence are given in Electronic Supplementary Material (ESM Fig. S1 and S2). All sequences were manually checked and consensus sequences were created (ESM Fig. S3).

BLAST alignment showed correspondence with both *Canis lupus* (accession code MK937053.1) and *Canis lupus familiaris* (accession code LR742875.1) mitochondrial genome, with an identity percentage of 99% for the hair entangled in the victim’s paws and of 100% for the hairs found in the victim’s mouth (ESM Fig. S4 and S5). It has to be noted that this mtDNA region is very well preserved and therefore identical between the sub-species *Canis lupus* (the wolf) and *Canis lupus familiaris* (the dog).

The results of the genetic analysis cleared foxes and nutrias as suspects, contrarily to the initial hypothesis. A wolf attack was considered very unlikely since wolves have never been reported in the area where the kill occurred. Besides, the type and the location of the wounds on the victim were inconsistent with the wolf killing pattern. The wolf is a specialized predator; its attack must be efficient, i.e. require as little energy as possible, and is usually aimed at eating. If the prey is small-sized, few, lethal wounds can be observed in the neck region of the animal and the carcass can be partially or entirely eaten. On the contrary, dogs lack the necessary experience for learning hunting techniques and predatory behaviour, which has a significant self-rewarding component. Consequently, a dog attack is characterized by the presence on the victim of numerous, non-lethal wounds all over the victim’s body: ears, face, neck, thorax, flanks, lateral and ventral abdomen, inguinal region, limbs, tail. Given the lower dog bite power, many bites result in contusions that can only be detected after skinning as sub-dermal haemorrhages and suffusions. Moreover, lacerations and tearing injuries due to the prey’s attempts to wriggle away are not uncommon and the carcass is not eaten [37].

Hence, we supposed that the attack was attributable to one or more medium- to large-sized dogs.

Performing canine STRs on swabs collected around the bitemarks would have been ideal to allow us to identify not only the species, but also the individual causing the bite marks. However, in this case, we were asked to intervene after the crime scene investigation and the carcass transfer to the diagnostic section; therefore, the carcass could have been exposed to external contamination. Even if we had sampled the areas around bite marks, we would not have had the certainty of the DNA source.

The morpho-metric features of the hairs collected from the victim’s oral cavity and paws corresponded to the hair coat length and colour of the landowner’s Hovawarts (black and gold, blond). This finding, together with the fact that the three Hovawarts were the only ones who had access to the property, pointed to them as prime suspects. In this specific case, we were not allowed to sample those dogs, and therefore, it was not possible to carry out further genetic investigations. Moreover, since the only reference sample in our possession consisted of shed hairs with no nuclear DNA, we could have only compared the mtDNA control region, whose exclusion capacity is, however, lower among dogs (normally ranging between 0.90 and 0.95) than humans (~ 0.995) and its discriminatory power restricted if compared with the genetic variation of nuclear DNA [19, 33, 38].

Notwithstanding these limitations, the cytochrome b gene analysis alone allowed us to unmask a false wild animal attack, thus putting the case in an entirely different perspective.

## Electronic supplementary material

ESM 1 (PDF 31926 kb).

## Data Availability

The datasets used and/or analysed during the current study are available from the corresponding author on reasonable request.
