# Supplementary material for: Who killed my dog? Use of forensic genetics to investigate an enigmatic case
Source: Int J Legal Med. 2020 Aug 11;135(2):387–92. doi: 10.1007/s00414-020-02388-9 (PMC7870635; doi:10.1007/s00414-020-02388-9)
Supplement: Supplementary file 1 — (PDF 31926 kb). [file 414_2020_2388_MOESM1_ESM.pdf]

ELECTRONIC SUPPLEMENTARY MATERIAL

Who killed my dog? Use of forensic genetics to investigate an enigmatic case

International Journal of Legal Medicine

Mariana Roccaro[1], Carla Bini[2], Paolo Fais[2], Giuseppe Merialdi[3], Susi Pelotti[2], Angelo Peli[1]

[1]Department of Veterinary Medical Sciences, University of Bologna, Ozzano dell’Emilia, Italy

[2]Department of Medical and Surgical Sciences, Unit of Legal Medicine, University of Bologna, Bologna, Italy

[3]Istituto Zooprofilattico Sperimentale della Lombardia e dell'Emilia–Romagna “Bruno Ubertini”, Bologna Unit, Bologna, Italy

Corresponding author: Mariana Roccaro

E-mail: mariana.roccaro2@unibo.it

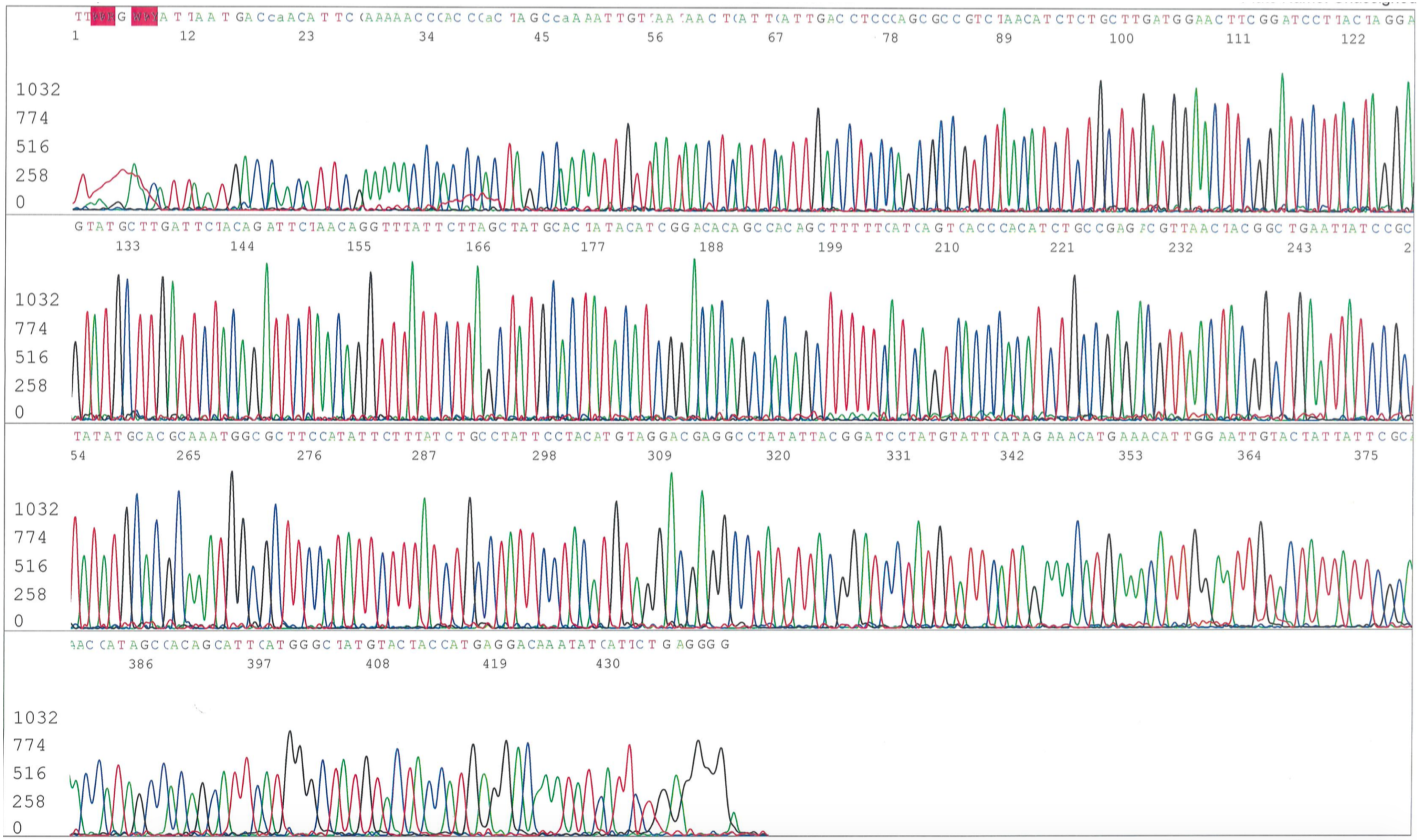

Fig. S1 Electropherogram of the CytB forward sequence from a hair sample entangled in the victim's mouth.

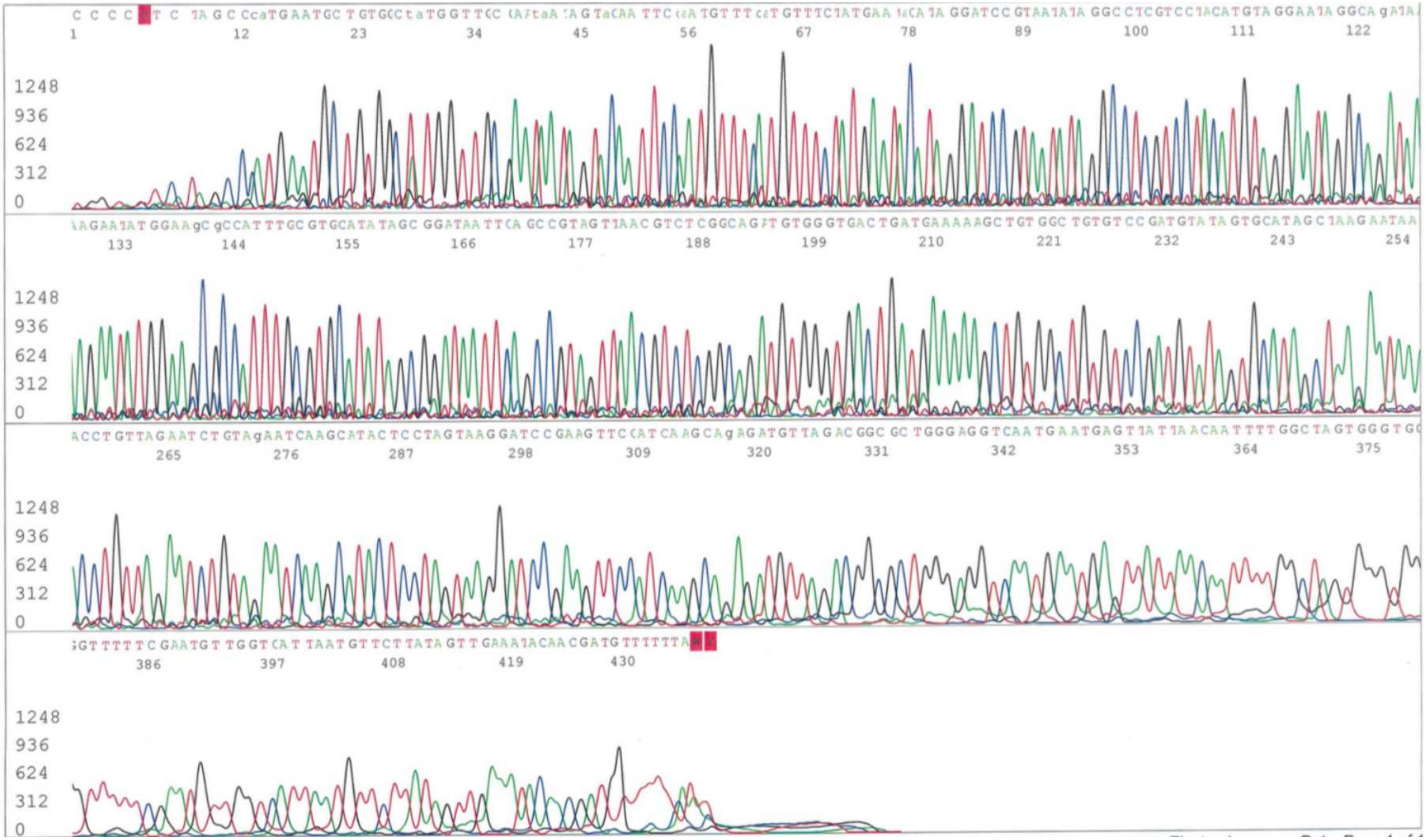

Fig. S2 Electropherogram of the CytB reverse sequence from a hair sample entangled in the victim's mouth.

CATCGTTGTATTTCAACTATAAGAACATTAATGACCAACATTCGAAAAACCCACCCACTAGCCAAAATTGTTAATAACTCATTCATTGACCTCC
CAGCGCCGTCTAACATCTCTGCTTGATGGAACTTCGGATCCTTACTAGGAGTATGCTTGATTCTACAGATTCTAACAGGTTTATTCTTAGCTATG
CACTATACATCGGACACAGCCACAGCTTTTTCATCAGTCACCCACATCTGCCGAGACGTTAACTACGGCTGAATTATCCGCTATATGCACGCAA
ATGGCGCTTCCATATTCTTTATCTGCCTATTCCTACATGTAGGACGAGGCCTATATTACGGATCCTATGTATTCATAGAAACATGAAACATTGGA
ATTGTACTATTATTCGCAACCATAGCCACAGCATTCATGGGCTATGTACTACCATGAGGACAAATATCATT

Fig. S3 Consensus sequence originated from the forward and reverse CytB sequences from a hair sample entangled in the victim's mouth.

Canis lupus isolate SK1_3x mitochondrion, partial genome

Sequence ID: MK937053.1 Length: 16430 Number of Matches: 1

Range 1: 14165 to 14613 GenBank Graphics ▼ Next Match ▲ Previous Match

Score	Expect	Identities	Gaps	Strand
830 bits(449)	0.0	449/449(100%)	0/449(0%)	Plus/Plus

```
Query  1      CATCGTTGTATTTCAACTATAAGAACATTAATGACCAACATTCGAAAAACCCACCCACTA  60
              ||||||||||||||||||||||||||||||||||||||||||||||||||||||||||||
Sbjct  14165  CATCGTTGTATTTCAACTATAAGAACATTAATGACCAACATTCGAAAAACCCACCCACTA  14224

Query  61     GCCAAAATTGTTAATAACTCATTCATTGACCTCCCAGCGCCGTCTAACATCTCTGCTTGA  120
              ||||||||||||||||||||||||||||||||||||||||||||||||||||||||||||
Sbjct  14225  GCCAAAATTGTTAATAACTCATTCATTGACCTCCCAGCGCCGTCTAACATCTCTGCTTGA  14284

Query  121    TGGAACTTCGGATCCTTACTAGGAGTATGCTTGATTCTACAGATTCTAACAGGTTTATTC  180
              ||||||||||||||||||||||||||||||||||||||||||||||||||||||||||||
Sbjct  14285  TGGAACTTCGGATCCTTACTAGGAGTATGCTTGATTCTACAGATTCTAACAGGTTTATTC  14344

Query  181    TTAGCTATGCACTATACATCGGACACAGCCACAGCTTTTTCATCAGTCACCCACATCTGC  240
              ||||||||||||||||||||||||||||||||||||||||||||||||||||||||||||
Sbjct  14345  TTAGCTATGCACTATACATCGGACACAGCCACAGCTTTTTCATCAGTCACCCACATCTGC  14404

Query  241    CGAGACGTTAACTACGGCTGAATTATCCGCTATATGCACGCAAATGGCGCTTCCATATTC  300
              ||||||||||||||||||||||||||||||||||||||||||||||||||||||||||||
Sbjct  14405  CGAGACGTTAACTACGGCTGAATTATCCGCTATATGCACGCAAATGGCGCTTCCATATTC  14464

Query  301    TTTATCTGCCTATTCCTACATGTAGGACGAGGCCTATATTACGGATCCTATGTATTCATA  360
              ||||||||||||||||||||||||||||||||||||||||||||||||||||||||||||
Sbjct  14465  TTTATCTGCCTATTCCTACATGTAGGACGAGGCCTATATTACGGATCCTATGTATTCATA  14524

Query  361    GAAACATGAAACATTGGAATTGTACTATTATTCGCAACCATAGCCACAGCATTCATGGGC  420
              ||||||||||||||||||||||||||||||||||||||||||||||||||||||||||||
Sbjct  14525  GAAACATGAAACATTGGAATTGTACTATTATTCGCAACCATAGCCACAGCATTCATGGGC  14584

Query  421    TATGTACTACCATGAGGACAAATATCATT  449
              |||||||||||||||||||||||||||||
Sbjct  14585  TATGTACTACCATGAGGACAAATATCATT  14613
```

Fig. S4 BLAST alignment results for the consensus CytB sequence from a hair sample entangled in the victim's mouth: *Canis lupus* mitochondrial genome (MK937053.1).

Canis lupus familiaris mitochondrial partial D-loop, isolate TRF.07.03_DMP

Sequence ID: LR742875.1 **Length:** 16124 **Number of Matches:** 1

Range 1: 14148 to 14596 GenBank Graphics ▼ Next Match ▲ Previous Match

Score	Expect	Identities	Gaps	Strand
830 bits(449)	0.0	449/449(100%)	0/449(0%)	Plus/Plus

```
Query  1      CATCGTTGTATTTCAACTATAAGAACATTAATGACCAACATTCGAAAAACCCACCCACTA  60
              ||||||||||||||||||||||||||||||||||||||||||||||||||||||||||||
Sbjct  14148  CATCGTTGTATTTCAACTATAAGAACATTAATGACCAACATTCGAAAAACCCACCCACTA  14207

Query  61     GCCAAAATTGTTAATAACTCATTCATTGACCTCCCAGCGCCGTCTAACATCTCTGCTTGA  120
              ||||||||||||||||||||||||||||||||||||||||||||||||||||||||||||
Sbjct  14208  GCCAAAATTGTTAATAACTCATTCATTGACCTCCCAGCGCCGTCTAACATCTCTGCTTGA  14267

Query  121    TGGAACTTCGGATCCTTACTAGGAGTATGCTTGATTCTACAGATTCTAACAGGTTTATTC  180
              ||||||||||||||||||||||||||||||||||||||||||||||||||||||||||||
Sbjct  14268  TGGAACTTCGGATCCTTACTAGGAGTATGCTTGATTCTACAGATTCTAACAGGTTTATTC  14327

Query  181    TTAGCTATGCACTATACATCGGACACAGCCACAGCTTTTTCATCAGTCACCCACATCTGC  240
              ||||||||||||||||||||||||||||||||||||||||||||||||||||||||||||
Sbjct  14328  TTAGCTATGCACTATACATCGGACACAGCCACAGCTTTTTCATCAGTCACCCACATCTGC  14387

Query  241    CGAGACGTTAACTACGGCTGAATTATCCGCTATATGCACGCAAATGGCGCTTCCATATTC  300
              ||||||||||||||||||||||||||||||||||||||||||||||||||||||||||||
Sbjct  14388  CGAGACGTTAACTACGGCTGAATTATCCGCTATATGCACGCAAATGGCGCTTCCATATTC  14447

Query  301    TTTATCTGCCTATTCCTACATGTAGGACGAGGCCTATATTACGGATCCTATGTATTCATA  360
              ||||||||||||||||||||||||||||||||||||||||||||||||||||||||||||
Sbjct  14448  TTTATCTGCCTATTCCTACATGTAGGACGAGGCCTATATTACGGATCCTATGTATTCATA  14507

Query  361    GAAACATGAAACATTGGAATTGTACTATTATTCGCAACCATAGCCACAGCATTCATGGGC  420
              ||||||||||||||||||||||||||||||||||||||||||||||||||||||||||||
Sbjct  14508  GAAACATGAAACATTGGAATTGTACTATTATTCGCAACCATAGCCACAGCATTCATGGGC  14567

Query  421    TATGTACTACCATGAGGACAAATATCATT  449
              |||||||||||||||||||||||||||||
Sbjct  14568  TATGTACTACCATGAGGACAAATATCATT  14596
```

Fig. S5 BLAST alignment results for the consensus CytB sequence from a hair sample entangled in the victim's mouth: *Canis lupus familiaris* mitochondrial genome (LR742875.1).
